# The Evolution of Hypertension Management in Canada: A Review of the Current Guidelines

**DOI:** 10.7759/cureus.99065

**Published:** 2025-12-12

**Authors:** Christina Cumaaran, Niharika Dahata, Rukayat O Balogun, Ifunanya C Modebelu, Dorcas A Adeola, Maureen O Obi, Uwanmwende D Omenai, Olamma A Dike, Michael Osunsedo, Oluchi C Abah

**Affiliations:** 1 Department of Internal Medicine, American International School of Medicine, Georgetown, GUY; 2 Department of Family Medicine, NHS England North East, Sunderland, GBR; 3 College of Medicine, University of Nigeria Teaching Hospital, Enugu, NGA; 4 Department of General Practice, Ladoke Akintola University of Technology, Ogbomosho, NGA; 5 Department of General Practice, Federal Teaching Hospital, Owerri, NGA; 6 Department of Psychiatry, Federal Neuropsychiatric Hospital, Benin, NGA; 7 Department of Family Medicine, Abia State University Teaching Hospital, Altona, CAN; 8 Department of Psychiatry, Priory Arnold, Nottingham, GBR; 9 Department of Family and Community Medicine, Abia State University, Uturu, NGA

**Keywords:** blood pressure control, canada, diet, exercise, guidelines, hypertension, lifestyle, medications, primary care

## Abstract

The burden of hypertension has increased in Canada over the last decade and remains a strong risk factor for cardiovascular morbidity and mortality. This has led Hypertension Canada to make it a public health priority and implement multiple strategies from different angles to solve this problem. The guidelines are taking a proactive and aggressive approach to bringing down the rates of hypertension. This narrative review aims to examine the evolution of Canadian hypertension guidelines from 2020 to 2025 and assess how the evidence has informed the updates for the new pharmacological and nonpharmacological interventions for adults over 18 years of age. The databases used were PubMed, Cochrane, and Google Scholar. The types of articles that were included were the Canadian National Hypertension Guidelines, reviews, controlled clinical trial data, and other relevant literature. Adult populations were included, and pediatric populations were excluded. The current guidelines have gained prominence in both clinical and policy discussions. Of note, four major findings were discovered in our review. They aim to have a concise but multidisciplinary approach to treating hypertension to achieve target treatment goals and reduce long-term disease outcomes. The new shift in the guidelines highlights lowering the threshold of treating hypertension at a blood pressure (BP) of ≤130/80 mmHg and the use of single-pill combination medication as first-line pharmacological therapy. Understanding these evolving trends is essential for clinicians and policymakers aiming to optimize patient outcomes and reduce the burden of cardiovascular disease (CVD).

## Introduction and background

Canada released new hypertension guidelines in 2025 to address the evolving needs of the emerging population. Twenty-one percent of adults in Alberta were diagnosed with hypertension in 2010 [1]. The average individual with hypertension had annual healthcare costs of $5768, of which 41% were attributed to hypertension, with 10.2% of the Canadian healthcare budget attributed solely to hypertension [1]. These are startlingly high numbers, and they are increasing every year, with a prediction that the situation will worsen. In the past five years, there have been systemic hurdles that Hypertension Canada has attempted to address with clinically proven treatment protocols based on the healthy lifestyle counseling, evidence-based treatment protocols, access to essential medicines and technology, risk-based cardiovascular disease (CVD) management, team-based care, and systems for monitoring (HEARTS) protocol developed by the World Health Organization (WHO) and the European and American hypertension guidelines.

Globally, hypertension is defined as systolic blood pressure (BP) of ≥140 mmHg or a diastolic blood pressure of ≥90 mmHg [2]. The treatment target in the most recent Canadian guidelines is systolic BP of ≤130 mmHg, with <120 mmHg considered for adults aged ≥50 years or those at elevated cardiovascular risk [3]. A landmark study, the 2015 SPRINT trial, has evidently demonstrated that achieving a systolic BP of <120 mmHg significantly reduced cardiovascular events and all-cause mortality in high-risk adults without diabetes [4]. Despite the updated guidelines, they still recommend the target threshold for individuals with diabetes to be ≤130/80 mmHg, and a threshold of <140/90 mmHg remains acceptable for lower-risk individuals [3]. These target goals have failed to be adequately reached globally. Canada was very proactive in controlling hypertension in the 2000s; by 2010, they had achieved a control rate of 68% compared to 13% in the 1990s [5]. However, the focus on hypertension fell on the back burner, which resulted in a drop from 69% to 49%, specifically in women [5]. In 2011, the nation put its effort to find a solution to this growing public health issue, but change has been slow to implement [5].

All of this shows how Canada has failed to address the modern-day challenges of hypertension. To mitigate this gap in our health system, Canada can implement improved screening, timely initiation of treatment, and consistent follow-up. The updated 2025 guidelines use a two-pronged approach to bridge this gap between evidence and practice by incorporating global frameworks such as HEARTS, clarifying blood pressure targets, and simplifying therapy initiation protocols [3]. The primary care component emphasizes real-world applicability and is designed to support clinicians in managing hypertension more effectively within routine practice. This aims to inform future policy in Canadian primary care.

## Review

Methods

This paper is a narrative review of the evolution of hypertension guidelines in Canada from 2020 to 2025. The databases used were PubMed, the Cochrane Library, and Google Scholar. The types of articles that were included are the Canadian National Hypertension Guidelines, reviews, controlled clinical trial data, and other relevant literature. Particular attention was paid to Canadian populations. Other international guidelines that were used to frame the Canadian guidelines were the European and American guidelines. The articles were assessed for full-text availability, and only free full-access articles were used. The search was limited to literature published from 2015 to 2025. Studies of adult and senior populations were included, along with studies of hypertension-associated comorbidities; pediatric populations were excluded. The keywords used were "Canadian," "hypertension," and "guidelines" and were combined using Boolean operators (AND and OR). A total of 1828 publications were initially identified. Nine hundred thirty-eight publications were removed after screening for full-text availability. The remaining full-text articles were further filtered based on title and abstract screening. The remaining 890 full-text articles were assessed for eligibility. Of the 890 full-text articles, 859 were excluded based on exclusion criteria. A total of 31 articles met the final inclusion criteria. Titles and abstracts were screened to identify sources that discussed Canadian hypertension guidelines or related updates. Articles that did not address the subjects were excluded.

Recent Canadian hypertension management and guidelines

Hypertension Canada released the recent 2025 guidelines. Hypertension is defined as a blood pressure value of ≥130/80 mmHg, with systolic pressure of >120 mmHg considered for adults aged ≥50 years of age or those at elevated cardiovascular risk. This is similar to the American guidelines (Table 1). American guidelines follow ≥130/80 mmHg, and pressures of >120/70 mmHg are considered for those at elevated cardiovascular risk. In contrast, the European guidelines follow ≥140/90 mmHg for the general population. While those with elevated cardiovascular risk, pressures of >120/70 mmHg are considered. It is evident that North American guidelines have a stricter threshold to define hypertension. Clearly, this is a growing problem in the population demographic and plays a role in metabolic syndrome and cardiovascular and heart diseases. These pose the greatest burden to the health system. Measuring blood pressure is done under guidelines with a validated device under optimal conditions [3]. A validated automated device is one that has been approved by Hypertension Canada using guidelines set by the Association for the Advancement of Medical Instrumentation (AAMI), the European Society of Hypertension (ESH), and the International Organization for Standardization (ISO) [3]. Validated devices are required to be used, along with a standardized method of measurement, as well as an out-of-office method to confirm the diagnosis of hypertension, or to detect white coat hypertension and masked hypertension [3].

**Table 1:** Comparison of Hypertension Targets Across Multiple Countries

Global	Canadian	American	European
≥140/90 mmHg	≥130/80 mmHg, with systolic pressure >120 mmHg considered for adults aged ≥50 years or those at elevated cardiovascular risk	≥130/80 mmHg and with pressures >120/70 mmHg considered for those at elevated cardiovascular risk	≥140/90 mmHg and with pressures >120/70 mmHg considered for those at elevated cardiovascular risk

First-line treatment starts with nonpharmacological intervention. Healthy lifestyle changes are prioritized for all adults with a treatment target of systolic blood pressure of ≤130 mmHg. Pharmacological interventions are initiated at a blood pressure of ≥140/90 mmHg or for adults with persistently elevated systolic blood pressure in the range of 130-139 mmHg with high cardiovascular risk. Single-pill combination (SPC) is the first drug of choice, with drugs from two of the three classes: angiotensin-converting enzyme inhibitors (ACEIs) or angiotensin receptor blockers (ARBs), thiazide or thiazide-like diuretics, and dihydropyridine calcium channel blockers (CCBs).

For nonpharmacological management, comprehensive lifestyle modifications are strongly recommended. These include a general overall weight reduction for overweight patients, adopting a heart-healthy diet, engaging in regular physical activity, achieving and maintaining a healthy body weight, quitting smoking, moderating alcohol consumption, and implementing effective stress management techniques [6]. These nonpharmacological measures not only support blood pressure control but also contribute to overall cardiovascular risk reduction [3,6].

Analysis of how medication choice and adjustments affect blood pressure control

To effectively manage high blood pressure, most adults with hypertension (approximately 70%) will require medications from more than one class of antihypertensives [3]. First-line therapies for adults with uncomplicated hypertension include ACEIs, ARBs, CCBs, and longer-acting thiazide-like diuretics [3]. β-Blockers are considered a first-line option only in younger patients without other complications [7]. Single-pill combination is the first drug of choice, with drugs from two of the three classes: angiotensin-converting enzyme inhibitors (ACEIs) or angiotensin receptor blockers (ARBs), thiazide or thiazide-like diuretics, and dihydropyridine calcium channel blockers (CCBs). SPCs improve patient outcomes by enhancing both adherence and tolerability. If there is limited availability or coverage does not include a single-pill combination, two separate pills can be substituted. The initial dosage is half a tablet of irbesartan/hydrochlorothiazide (HCTZ) 300/25 mg. If the target is not reached, the dose is increased to one full tablet of irbesartan/HCTZ 300/25 mg. If blood pressure remains high, amlodipine 5 mg daily can be started and increased to 10 mg daily. Finally, spironolactone 12.5 mg titrated up to 25 mg can be added for hypertension resistant to treatment, and/or a referral to specialists can be done for secondary concerns of hypertension, as the final steps in primary care treatment [3].

As the updated blood pressure thresholds in the guidelines lower the definition and targets for hypertension, more individuals will now fall under the diagnosis. This has potential implications, such as stigma or challenges with insurance. Nevertheless, the shift toward earlier detection and treatment is supported by robust evidence and is expected to reduce long-term cardiovascular risks on a population level [6].

Individuals with an elevated risk of cardiovascular disease are managed more aggressively when it comes to blood pressure control. For these patients, antihypertensive therapy is typically initiated at a lower threshold, specifically when systolic blood pressure is >130 mmHg or diastolic pressure is >80 mmHg. The treatment goal is also more stringent, with a target blood pressure often set at <120/80 mmHg to reduce the risk of cardiovascular events such as heart attack and stroke (Table 2) [3].

**Table 2:** Comparison of Hypertension Guidelines in Canada From 2020 to 2025 CVD, cardiovascular disease; SBP, systolic blood pressure; DBP, diastolic blood pressure

Patient population	Old	New
Low risk (no CVD)	SBP < 140; DBP < 90	≥130/80
Risk of CVD	SBP < 120	SBP < 130
Diabetes	SBP < 130; DBP < 90	SBP < 130

The most efficient treatment outlined in the new guidelines is single-dose formulations to address all of these target goals. When medications are combined into a single-dose formulation, they often demonstrate 65% enhanced effectiveness and fewer side effects, even at lower individual doses, compared to 48% effectiveness using multiple medications separately [3]. Evidence from meta-analyses indicates that SPCs reduce systolic blood pressure by an average of 4.0 mmHg, compared to free-equivalent drug combinations [3].

Adherence is also notably better with SPCs, with potential benefits in long-term persistence. Data from more than 100000 patients shows that beginning with combination therapy helps patients stay on effective multidrug treatments in the long term, being over twice as likely to continue them after three years, compared to those who started on a single medication [3].

Usually, cost is a deterrent to drug treatment adherence. Luckily, SPCs happen to be more affordable than purchasing each drug separately, bypassing this issue. A Canadian study from 2009 estimated that these combinations could save more annually in medication costs alone. Importantly, clinical trial data has not shown higher rates of treatment discontinuation due to side effects with SPCs, compared to either free-drug combinations or monotherapy. However, dizziness appears more frequently with SPCs than with standard-dose monotherapy [3].

Another compounding challenge is in the elderly population. Older patients often face complex medication schedules. In these older populations, pharmacist-led interventions have played a critical role in advancing safe medication reduction strategies. Canadian studies such as Eliminating Medications Through Patient Ownership of End Results (EMPOWER) and Developing Pharmacist-led Research to Educate and Sensitize Community Residents to the Inappropriate Prescription Burden in the Elderly (D-PRESCRIBE), which focus on pharmacist-led deprescribing of sedatives, have shown that these interventions are effective and feasible. Reducing pill burden through the use of SPCs or longer-acting medications, such as lisinopril, instead of captopril, can enhance both adherence and blood pressure control [3]. These findings are particularly relevant for elderly patients with hypertension, where minimizing unnecessary medications, such as β-blockers or α-blockers, can help reduce fall risk and adverse drug effects.

Impact of diet on blood pressure outcomes in Canada

In Canadian primary care, dietary and lifestyle changes continue to be essential interventions for the prevention and treatment of hypertension, supplementing pharmacological treatment when necessary. Current WHO guidelines strongly recommend dietary changes for all adults with hypertension, with high certainty of evidence for improved outcomes [2,3,6,8-10].

Among these, the implementation of dietary interventions, such as the Dietary Approaches to Stop Hypertension (DASH) diet, sodium reduction, limiting processed food intake, and encouraging potassium-rich food consumption, has shown favorable effects on blood pressure control [3]. The DASH diet, rich in fruits, vegetables, whole grains, and low-fat dairy, has been repeatedly endorsed by Hypertension Canada due to its consistent impact on lowering systolic and diastolic blood pressure, especially when combined with sodium restriction [3]. Among individuals 60 years of age or older with hypertension and a history of stroke, lowering dietary sodium intake to below 2 g per day in adults with a salt substitute resulted in a 13% reduction in major adverse cardiovascular events and a 12% reduction in all-cause death over approximately five years [3]. Despite these recommendations, average sodium consumption in Canada remains above this threshold, largely due to the high intake of processed and prepackaged foods. Interestingly, processed foods rather than table salt account for about 70% of the sodium intake from the average Canadian diet, which suggests that upstream regulatory and food industry reforms are necessary [8]. The C-CHANGE guidelines emphasize that population-wide blood pressure regulation requires addressing dietary factors through patient education and system-level interventions (such as food labeling and sodium targets for manufacturers) [6].

Despite being naturally abundant in fruits and vegetables, potassium, a crucial nutrient in blood pressure regulation, is frequently under-consumed, particularly among older adults and low-income populations, due to affordability and accessibility issues [10]. To reduce the burden of hypertension and related diseases, the WHO recommends minimizing the average population's daily sodium intake while increasing potassium intake to at least 3.5 g daily [9]. This consequently lowers cardiovascular risk and counteracts sodium-induced blood pressure elevations [2]. However, potassium levels should be cautiously monitored in individuals suffering from chronic kidney disease or those taking medications that may increase blood potassium levels, such as mineralocorticoid receptor antagonists, angiotensin II receptor blockers (ARBs), and angiotensin-converting enzyme inhibitors (ACEIs) [2].

There is growing acceptance of low-sodium salt substitutes (LSSS) as a potential way to reduce sodium intake. Interventional studies have linked LSSS to marginal reductions in blood pressure and cardiovascular events [10]. Although evidence of the benefits of LSSS in the Canadian context is still emerging, there is the potential for wide adoption and success, particularly when targeted to high-risk groups. This makes it possible for potassium-containing LSSS, or other comparable minerals, to increase potassium intake while decreasing salt consumption at the same time [10].

Multicomponent approaches are becoming more common in clinical and community settings in Canada as another way to address diet in the treatment of hypertension. This is consistent with the HEARTS framework developed by the WHO, which seeks to integrate these suggestions into effective, practical, and evidence-based algorithms for use in Canadian primary care [2]. For example, evaluations of primary care teams in Canada have shown improved adherence to evidence-based chronic disease management guidelines [2]. The C-CHANGE 2022 guideline also implements this approach across multiple chronic diseases (including hypertension) by promoting integrated lifestyle, pharmacological, and risk-based interventions in primary care settings [6]. Dietary counseling with the incorporation of nutritional interventions into primary care and the use of technology, such as mobile applications for dietary monitoring, are major recently implemented strategies in Canadian community and clinic settings [2,3]. These interventions have drastically improved hypertension results. The RxPATH randomized study, which led to a 4.76 mmHg greater reduction in systolic blood pressure over three months compared to usual care, exemplifies how certification programs that operationalize guideline-driven protocols can support WHO's strategic vision for hypertension management [2].

How the role of exercise and activity affects the change in blood pressure regulation

The 2025 guidelines made exercise and physical activity the forefront of nonpharmacological intervention. Moderate-intensity aerobic exercise, such as walking, jogging, cycling, or swimming, in addition to routine activities of daily living, is recommended. For hypertensive individuals, doing exercise for 150-300 minutes weekly has shown an improvement in mean systolic and diastolic blood pressure by 6.9 mmHg and 4.9 mmHg, respectively [3,11]. This has been proven to be a direct correlation in the Canadian Longitudinal Study on Aging (CLSA) [12]. This effect of physical activity has particular importance in older adults, as it has shown evidence to mediate the relationship between frailty and hypertension [13]. Collectively, the data reinforces that prioritizing exercise is clinically effective in managing hypertension from a nonpharmacological standpoint.

When adding the intersectionality of living situations, whether in urban or rural settings, along with physical activity, more nuances need to be taken into account. Between these different regional populations, exercise habits and sedentary behavior vary. Urban residents often have greater access to fitness facilities, walkable infrastructure, and public health campaigns promoting physical activity, which can help manage hypertension [3]. Conflicting to this, dense urban settings can often lack accessible green spaces or facilities that promote movement, further reinforcing sedentary lifestyles. Adults in the urban setting spend an average of 9.8 hours/day in sedentary activities, such as commuting, desk-bound jobs, and screen time, potentially offsetting these benefits [14].

This trend may be more pronounced in rural areas, as they have limited access to healthcare and lack access to recreational spaces, as well as less active transportation options. This prolonged inactivity is a recognized risk factor for hypertension and cardiovascular strain [13]. There is also a higher prevalence of systolic blood pressure in residents of rural areas, even after adjusting for their living environments [15,16].

Addressing these lifestyle factors is crucial to decreasing the issues in hypertension treatment. The Canadian 24-hour movement guidelines emphasize limiting sedentary time to eight hours or less daily to mitigate these risks and support cardiovascular health [13]. Currently, only 26.5% of individuals actually meet these recommended thresholds [14]. A range of physical activity is encouraged, such as weight-bearing, sports, and recreational activities in a variety of environments, such as home, work, community, indoor/outdoor, land/water, and across all contexts. Physical activity helps with not only hypertension but also a vast number of comorbidities and chronic diseases, such as the decreased incidence of type 2 diabetes, anxiety, depression, and several cancers. This may be a challenge in Canada, as winters are cold and long. Resistance training has been shown to have a particularly strong impact on lowering blood pressure and reducing the incidence of cardiovascular disease and myocardial infarction. These guidelines also emphasize the importance of sleep hygiene; they recommend 7-9 hours of high-quality sleep. Sleep usually gets overlooked, but according to recommendations, it plays a vital role in so many diseases, including affecting blood pressure [13].

To add to the 24-hour movement guidelines, two other novel approaches are being used to attack the situation. Primarily, there is the PaRx program, adapted from the Green Prescription program in the United States, which encourages time out in nature [17,18]. It was inspired by an attempt to decentralize Western practices and utilize Indigenous communities' ideology of prioritizing land-based healing practices [18]. Healthcare providers are allowed to formally prescribe patients to go out in nature, which in turn promotes stress reduction, increased physical activity, and overall cardiovascular health [18]. A positive side effect is that it also fosters improved mental health and reduced isolation and loneliness [18]. The earliest province to adopt this program was Ontario in 2018, and several other provinces have followed suit over the years [18]. The second approach is wearable technology. Wearable devices give patients the ability to accurately track and identify trends with continuous home monitoring, enabling doctors to make more informed treatment changes in conjunction with their patients [3]. This two-pronged approach uses both community engagement and digital health tools to offer a scalable model for improving hypertension outcomes across diverse Canadian populations.

Linking guidelines to clinical practice

Even though guidelines might be in place, implementing them in real-world settings is another matter entirely. One challenge is due to the frequent updates to the hypertension guidelines; a survey of Canadian physicians showed that the enthusiasm to adhere to them is short of expectations [19]. Barriers to successfully implementing the guidelines can be identified at the level of the clinician, the patient, and the practice setting. They include discordance between guidelines produced by different organizations, failures to address clinically relevant issues, failures to incorporate patient-clinician values, and a lack of local involvement [19]. On the part of the clinician, the lack of outcome expectancy, familiarity, motivation, and self-efficacy are part of the barriers [19]. On the part of the patient, their preferences may contradict guidelines and may prevent doctors from treating them effectively [20]. These encounters between the physician and patient, in real-world practice settings, have to be tailored for the patients' individual needs and their autonomy while also providing quality care on the part of the physician.

The guidelines demonstrate that the pills have superior efficacy for reaching target treatment goals [3]. In people with mild hypertension who only require dietary changes as a first-line intervention, they actually prefer to skip this first-line intervention and would rather take the pill. However, the younger, lower-risk population seems to disagree with this sentiment. They are more inclined to manage their condition through diet rather than pharmacotherapy [20].

Another facet to integrating recommendations into clinical practice is proper access to healthcare, such as income, occupation, education, and housing. They are a major reason why some groups face worse health outcomes than others. These factors are responsible for 30%-55% of health outcomes, far outweighing the impact of personal choices such as diet or exercise [21]. This stems from low access to healthcare and low transportation in rural areas [22]. Around 40% of adults in rural areas are living with high blood pressure, compared to just 29% in urban areas [22]. When it comes to low-income groups, 50% of individuals were seen not to be adherent to medication, contributing to suboptimal hypertension control, even though Canada has universal healthcare [13]. Living on a low income is closely tied to higher rates of illness and disability. It is a clear example of how health and social conditions are deeply connected and how poverty itself becomes both a cause and a consequence of poor health [21].

Indigenous peoples have held on to their culture, values, and ways of life, but even today, many Western systems, especially healthcare, still carry policies and practices that do more harm than good. Due to this, they often face much worse health outcomes than others, including some of the highest rates of heart disease and early death [23]. Wali et al. reported that in Canada, First Nations men have a 30% higher death rate from heart disease than other men, and for First Nations women, it is 76% higher [21]. These higher rates are closely linked to long-standing inequalities in living conditions such as housing, income, food access, and experiences of racism that affect health across generations. All of these factors, compounded with traumatic experiences in their lifetime, increase the risk of cardiovascular disease (CVD). This contributes to the negative impacts on the health of the Indigenous community [23].

Limitations and future directions

This narrative review has a selective bias limitation, as a limited number of articles were selected for the discussion, thus leading to potential bias, limited generalizability, and the lack of quantifiable evidence. This narrative review did not include a formal systematic assessment of study quality or risk of bias. As a result, the strength of the supporting evidence may vary, and this should be considered when interpreting the conclusion. Most of the data obtained for the guidelines included limited Canadian populations, as most of it is based on American and European guidelines. Additionally, the research into Canadian hypertension fails to include sufficient data from Indigenous communities, rural communities, and immigrant groups, resulting in sampling bias and diminishing the strength of the evidence [2,24]. Public healthcare in the nation at large needs to develop cultural programs to enhance blood pressure management in the vast diaspora that is the Canadian population. In particular, because of the lack of trust between Indigenous people and the healthcare system, these programs need to be endorsed by trusted community leaders to execute change [21]. Examples of culturally appropriate activities that could help integrate healthy lifestyle changes include food harvesting, traditional dancing and ceremonial practices, and land-based learning.

The previous guidelines were also confusing; thus, their effectiveness in creating long-term sustainable treatment outcomes was low. Moreover, Canada lacks proper national implementation of interdisciplinary teams, on top of having limited durations for patient visit times [5]. The lack of doctors and the increased number of patients have decreased one-on-one time per patient visit [5]. This gap in care has resulted in many Canadians not having family doctors for ongoing care. They end up trickling down into emergency centers, urgent care, and walk-in clinics. This limited continuous care creates challenges for the management of patients. The electronic medical record (EMR) between provinces is also not at a national level, which decreases the reliability of the application of guidelines and the continual surveillance of treatment [25]. In the United Kingdom, it has been demonstrated that the utilization of clinical reminder systems improved patient adherence to chronic medication treatments [26]. Canada could implement this in their EMR systems to do the same. Blood pressure results also improve when healthcare teams consisting of physicians, pharmacists, dietitians, and nurse practitioners work together.

Lastly, since the new guidelines were only implemented this year, there is insufficient data on how the new classes of medications will affect the treatment and management of hypertension. The guidelines also do not account for complex cases and causes of hypertension, which are becoming more prevalent in Canada as the population ages.

## Conclusions

Compared to the rest of the world, Canada has created a more stringent guideline to deal with the increasing rates of hypertension nationally. They have taken actionable, concrete steps to achieve a well-rounded approach to address hypertension as a chronic disease. They are addressing not only the drug aspect but also the person as a whole, how psychological, social, and behavioral aspects all need to be taken into account to effectively treat a patient. This act of balancing all of these factors cannot rely only on one facet of the healthcare system; the mosaic of Canada as a whole has to take on this responsibility.
